# Rapid evolution of a bacterial parasite during outbreaks in two *Daphnia* populations

**DOI:** 10.1002/ece3.9676

**Published:** 2023-01-16

**Authors:** Clara L. Shaw, Meghan A. Duffy

**Affiliations:** ^1^ Department of Ecology & Evolutionary Biology University of Michigan Ann Arbor Michigan USA; ^2^ Department of Biology The Pennsylvania State University University Park Pennsylvania USA

**Keywords:** *Daphnia*, epidemic, evolution, matching alleles, parasite, *Pasteuria ramosa*

## Abstract

Myriad ecological and evolutionary factors can influence whether a particular parasite successfully transmits to a new host during a disease outbreak, with consequences for the structure and diversity of parasite populations. However, even though the diversity and evolution of parasite populations are of clear fundamental and applied importance, we have surprisingly few studies that track how genetic structure of parasites changes during naturally occurring outbreaks in non‐human populations. Here, we used population genetic approaches to reveal how genotypes of a bacterial parasite, *Pasteuria ramosa,* change over time, focusing on how infecting *P. ramosa* genotypes change during the course of epidemics in *Daphnia* populations in two lakes. We found evidence for genetic change – and, therefore, evolution – of the parasite during outbreaks. In one lake, *P. ramosa* genotypes were structured by sampling date; in both lakes, genetic distance between groups of *P. ramosa* isolates increased with time between sampling. Diversity in parasite populations remained constant over epidemics, although one epidemic (which was large) had low genetic diversity while the other epidemic (which was small) had high genetic diversity. Our findings demonstrate that patterns of parasite evolution differ between outbreaks; future studies exploring the feedbacks among epidemic size, host diversity, and parasite genetic diversity would improve our understanding of parasite dynamics and evolution.

## Graphical abstract

We examined the genetic structure and diversity of the parasite *Pasteuria ramosa* in two natural outbreaks using variable number tandem repeats to genotype the parasite in infected *Daphnia dentifera* hosts. We found evidence for parasite evolution within these outbreaks: the genetic distance between sampled parasite genotypes infecting hosts increased with time between sampling, but with notable differences between the two lakes. Although genotype diversity remained flat over time in both outbreaks, it was significantly higher in the lake with the smaller outbreak. This work is valuable for understanding evolution and patterns of parasite genetic diversity within outbreaks.

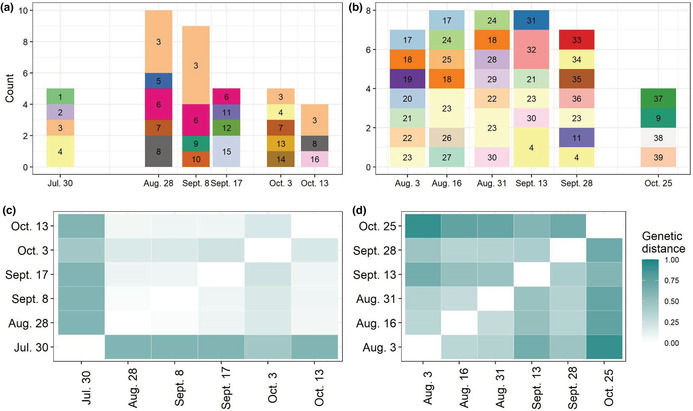

## 1 INTRODUCTION

Parasite genotypes vary in traits that impact their fitness, including infectivity, virulence, and the ability to persist in the environment (Refardt & Ebert, 2007; Rogalski & Duffy, 2020; Salvaudon et al., 2005) As epidemics progress, parasite population structure changes due to selection on these traits, which can influence epidemic dynamics. Understanding how parasite population structure and diversity change during epidemics is important for public health, conservation, and our fundamental understanding of parasitism. Despite this, aside from studies on some humans pathogens (e.g., ebolavirus (Park et al., 2015), influenza (McCrone et al., 2018), and SARS‐CoV‐2 (Forster et al., 2020; Lin et al., 2021)), few empirical studies have analyzed changes in parasite population structure and diversity during natural epidemics in wild systems, particularly in systems where multiple ecological conditions can be explored (but see Eck et al., 2021; Zhan et al., 2002). This gap leaves us without a clear understanding of parasite evolution during epidemics in wild hosts, or how different ecological scenarios might influence changes in parasite population structure and diversity.

Predicting how population structure and diversity will change over time is challenging for several reasons (Burdon, 1993). First, the assemblage of hosts is an important selective force on parasites (Gandon, 2004; Koskella, 2014; Paplauskas et al., 2021). However, hosts could impose selection that decreases diversity (if selection is directional) or maintains and even increases it if the most common parasite genotypes are less successful and rare genotypes are more successful (i.e., negative frequency‐dependent selection). Second, the greater ecology of a system, where there are myriad interactions among hosts, parasites, and their biotic and abiotic environment, may impact selection in epidemics in the wild (Paplauskas et al., 2021). Third, chance events could impact parasite genotype frequencies due to bottlenecks and rapid changes in parasite population sizes (Papkou et al., 2016). Fourth, starting parasite diversity is an important determinant of parasite evolution (Eck et al., 2021), but the amount of variation at the start of an epidemic might vary substantially (e.g., if the epidemic started due to the introduction of a few infected migrants vs. being triggered by contact between the host population and large numbers of persistent environmental transmission stages in a spore bank). Given the diversity of factors that might influence parasite evolution and the paucity of prior studies monitoring changes in parasite population structure and diversity over the course of an outbreak, we still lack an understanding of how parasite assemblages change over time, or even whether genetic diversity tends to increase, decrease, or stay consistent during epidemics.

We studied how genotypes of the widespread bacterial parasite *Pasteuria ramosa* changed during two natural outbreaks in *Daphnia dentifera* hosts, important planktonic grazers in lakes. Hosts become infected after consuming *P. ramosa* environmental transmission stages (spores) floating in the water column. Infection requires attachment of spores to the host esophagus (Duneau et al., 2011) as well as evasion of additional within‐host processes that can prevent parasite infection and proliferation after the attachment step (Luijckx et al., 2014). Resistance to spore attachment is governed by multiple alleles at one locus in the host, giving rise to a matching‐allele model of infection (Bento et al., 2017; Routtu & Ebert, 2015). After infection, the parasite sterilizes its host (preventing a host genotype to which it is infective from producing more susceptible progeny) and propagates itself within the host hemolymph (Ebert et al., 1996). *P. ramosa* is an obligate killer, and spores are only released from decaying host corpses (Ebert et al., 1996). These spores can go on to infect susceptible hosts, and thereby extend an outbreak, or remain infective for many decades in lake sediments (Decaestecker et al., 2004, 2007). Within an epidemic season, uninfected *D. dentifera* hosts reproduce asexually, yielding many asexual clutches (Smirnov, 2017), only switching to sexual reproduction late in the fall toward the end of epidemics (Duffy et al., 2008; Gowler et al., 2021; Hite et al., 2017). Sexual offspring are enclosed in resting eggs that overwinter in sediments. Therefore, host diversity during an epidemic is governed by evolutionary forces acting on standing variation in hosts after sexual offspring hatch in the spring. We hypothesized that parasite diversity during an epidemic is similarly governed by evolutionary forces acting on standing variation that had been seeded from the spore bank.

Although there is phenotypic evidence that *P. ramosa* evolution occurs over the course of outbreaks (Auld et al., 2014; Gowler et al., 2022; Paplauskas et al., 2021), we did not know how parasite genotype assemblages would change within outbreaks. Parasite genotypic diversity could increase as mutations occur, decrease if parasites adapt to low‐diversity host populations, or be maintained if different genotypes are favored through time due to negative frequency‐dependent selection and/or by migration from other lakes or reintroduction from the spore bank. We tracked genotypes of *P. ramosa* that successfully infected hosts during outbreaks in two lakes and used variable number tandem repeats (VNTRs) to assess how *P. ramosa* genotype assemblages changed during natural outbreaks (Andras & Ebert, 2013; Mouton & Ebert, 2008).

## 2 METHODS

We tracked epidemics in two lakes in southeast Michigan and collected infected hosts from multiple time points in these epidemics to track parasite diversity over time. The lakes, Little Appleton (Waterloo Recreation Area) and Crooked Lake (Pinckney Recreation Area; known as “Crooked‐P” in other publications on this *Daphnia*–parasite system to distinguish it from another Crooked Lake), were sampled every 2 weeks from mid‐July until mid‐November 2017 by combining three plankton tows (using a 12 cm Wisconsin net, 153 μm) from at least 10 m apart at the deepest part of each lake. Two samples, each combining three plankton tows from the deep basin, were collected by this method; the first sample was used to assess infection prevalence and the second was used to measure host density. For infection prevalence, subsamples from one of these combined samples were taken and hosts were counted and visually diagnosed for *P. ramosa* infection using a dissecting microscope until at least 200 *D. dentifera* individuals were counted or until the entire sample was processed. While processing, we collected infected hosts and preserved them individually in 90% ethanol. Preserved infected hosts were stored at −20°C until DNA extraction. The second combined sample was preserved in 90% ethanol and later subsampled volumetrically and counted under a dissecting microscope to assess host density. Infected host density was calculated by multiplying infection prevalence by host density at each sample date.

Genotyping parasites required DNA extraction from infected animals, PCR amplification of VNTRs, and analysis of VNTR lengths via fragment analysis. For DNA extraction, preserved infected animals were removed from ethanol and placed in sterile microcentrifuge tubes. We used the mericon bacteria plus DNA extraction kit (Qiagen, Hilden, Germany) to extract DNA. The preserved infected animals were vortexed in 200 μl fast lysis buffer with a battery‐powered pestle to make an emulsion. Emulsions were transferred to “pathogen lysis” tubes and vortexed for 10 minutes. These tubes were then centrifuged at 11,500 rpm. The DNA‐containing supernatant was removed and saved. We attempted to amplify samples at 11 VNTR loci by PCR (Andras & Ebert, 2013; Mouton & Ebert, 2008; Appendix S1). We carried out reactions in 10 μl volumes of 1X Qiagen multiplex mastermix (QIAGEN), 10 nM forward primer with M13(−21) tail, 400 nM reverse primer, and 400 nM M13(−21) 6FAM‐labeled forward primer or M13(−21) HEX‐labeled forward primer. The labeled primers allowed loci to be identified in fragment analysis (Schuelke, 2000). Amplification conditions were as follows: 94°C (15 min), then 42 cycles of 94°C (30 s)/50°C (30 s)/72°C (1 min), and a final extension time at 72°C for 10 min. Following PCR, loci with distinct labels were combined and diluted 1:50 in molecular‐grade water. One microliter diluted product was added into capillary electrophoresis loading plates containing 1 μl Hi‐Di formamide and a LIZ500 or a ROX500 size standard (University of Michigan DNA Sequencing Core). Fragment analysis was performed by the University of Michigan DNA sequencing core. We used the software GeneMapper (ThermoFisher Scientific) to read fragment lengths.

We analyzed parasite genotypes (combined alleles at all VNTR loci) to quantify parasite diversity in the two lakes and to understand how parasite genotype structure and diversity changed over time in epidemics. In our analysis, we excluded three loci: Pr17 because it was uniform across all samples and Pr3 and Pr7 due to poor amplification (missing in 39.3% and 28.6% of samples, respectively). This left eight loci in our analysis. We subsequently removed from analysis any samples without amplification for at least six of the eight remaining VNTR loci. Samples were assigned to the same multilocus genotype (MLG) if they were identical at all loci (including loci with missing data—loci with missing data were assumed to have null alleles). In several samples (3 of the 38 Little Appleton samples and 2 of the 42 Crooked samples), multiple alleles were present for a given locus, indicating coinfection with multiple *P. ramosa* genotypes. We thus created two datasets for each lake: one using the alleles with the highest amplification in coinfected animals (i.e., ignoring coinfection), and another that included two MLGs within infected animals. For the dataset that included coinfections, alleles that were secondary in amplification were assumed to belong to the same coinfecting MLG; when only one allele was amplified within a coinfected host at a particular locus that allele was assumed to be the allele for both coinfecting genotypes. Analysis with this coinfection dataset yielded qualitatively similar results to the dataset with a single genotype identified per host; we therefore only present analysis from the latter.

Three metrics were used to quantify how parasite genotype diversity was structured and changed over time: comparisons of Nei's gene diversity between lakes and sampling dates, analysis of molecular variance (AMOVA), and Prevosti distances between parasite genotype assemblages at different sampling dates within lakes. We first calculated Nei's gene diversity at each sample date for each lake (Nei, 1973). This metric measures the probability that two randomly drawn alleles for a given locus will be different from each other (Nei, 1973). We bootstrapped values of Nei's gene diversity, resampling 1000 times, and centered confidence intervals around the observed values (Marcon et al., 2012). We then used linear models to assess changes in Nei's gene diversity over time, and we used a *t*‐test to compare levels of gene diversity between lakes. Second, to quantify the extent to which parasite genotypes structured by sampling date, we constructed a Prevosti distance matrix (i.e., the fraction of allelic differences out of all loci (Wright, 1978) between all parasite samples) and performed an AMOVA for each lake partitioning by sample date where parasite assemblages sampled at different dates were treated independently (Excoffier et al., 1992). This analysis is analogous to an analysis of variance where the variation that is partitioned are the genetic distances between pairs of individuals. Significance of the partitioning is then determined by randomly permuting the distance matrix (in our case, 1000 times), each time calculating variance assigned to sample dates to create a null distribution for comparison (Excoffier et al., 1992). Lastly, we calculated Prevosti distance (absolute genetic distance) between parasite populations from different sample dates for each lake (Prevosti et al., 1975). In contrast to the Prevosti distance metric between individuals described above, this metric measures the average difference in allele frequencies over all loci between two assemblages (here, sample dates). We used a linear model to test if genetic distance between parasite assemblages was related to the amount of time that had passed between sampling dates. All statistical tests were performed in R version 4.0.3 (R Core Team, 2020). All population genetics calculations were computed using the R package “Poppr” version 2.9.3 (Kamvar et al., 2014). All figures were constructed using the R package “ggplot2” version 3.3.5 (Wickham, 2016).

## 3 RESULTS

**FIGURE 1:**
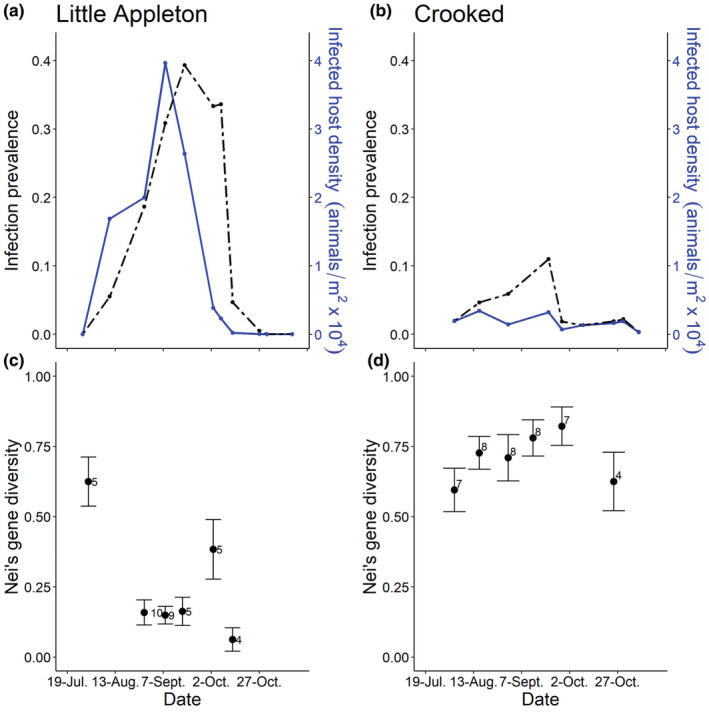
Crooked had a smaller but more diverse outbreak than Little Appleton. (a and b) Outbreak size was much larger in Little Appleton than in Crooked both in terms of infection prevalence (left axis, dashed black line) and infected host density (right axis, solid blue line). (c and d) There was no clear pattern of changing gene diversity over time in Little Appleton (panel c) or Crooked (panel d). Gene diversity was higher in Crooked than in Little Appleton when all sample dates were considered. Sample sizes are noted next to points; error bars show centered bootstrapped 95% confidence intervals (Marcon et al., 2012).

The outbreak in Little Appleton was much larger than the outbreak in Crooked in terms of both infection prevalence and infected host density (Figure 1a,b). Despite the substantially larger numbers of infected hosts, both allelic and MLG diversity were lower in Little Appleton than in Crooked with an average of 3.62 alleles per locus in Little Appleton and 6.38 alleles per locus in Crooked; over the course of the outbreak, there were only 16 MLGs in Little Appleton (of 38 samples) compared to 26 MLGs in Crooked (of 42 samples). Nei's gene diversity remained flat over time in both outbreaks (Little Appleton: *F*_1,4_ = 2.86, *p* = .17; Crooked: *F*_1,4_ = 0.13, *p* = .74) although it was higher in Crooked (*t* = 4.89, *p* = .002; Figure 1c,d). In one lake, Little Appleton, parasite genotypes were structured by sample date (Table 1), with parasites collected on a given date being more similar to one another than they were to parasites collected on a different date. In contrast, date did not have a significant effect in Crooked (Table 1).

**TABLE 1:** Results of AMOVA on structure over time.

	Variance	Percent of total variance	*p* value^a^	ɸ
Little Appleton	Between dates: 0.50 Within dates: 3.87	Between dates: 11.48% Within dates: 88.52%	*p* = .011	ɸ_dates‐total_ = 0.11
Crooked	Between dates: 0.27 Within dates: 6.88	Between dates: 3.83% Within dates: 96.17%	*p* = .173	ɸ_dates‐total_ = 0.04

^a^ *p* value based on comparison of variance components with null distribution created with 1000 random permutations of the distance matrix. In both lakes, the variation between sampling dates was greater than expected by chance but only significantly greater in Little Appleton.

**FIGURE 2:**
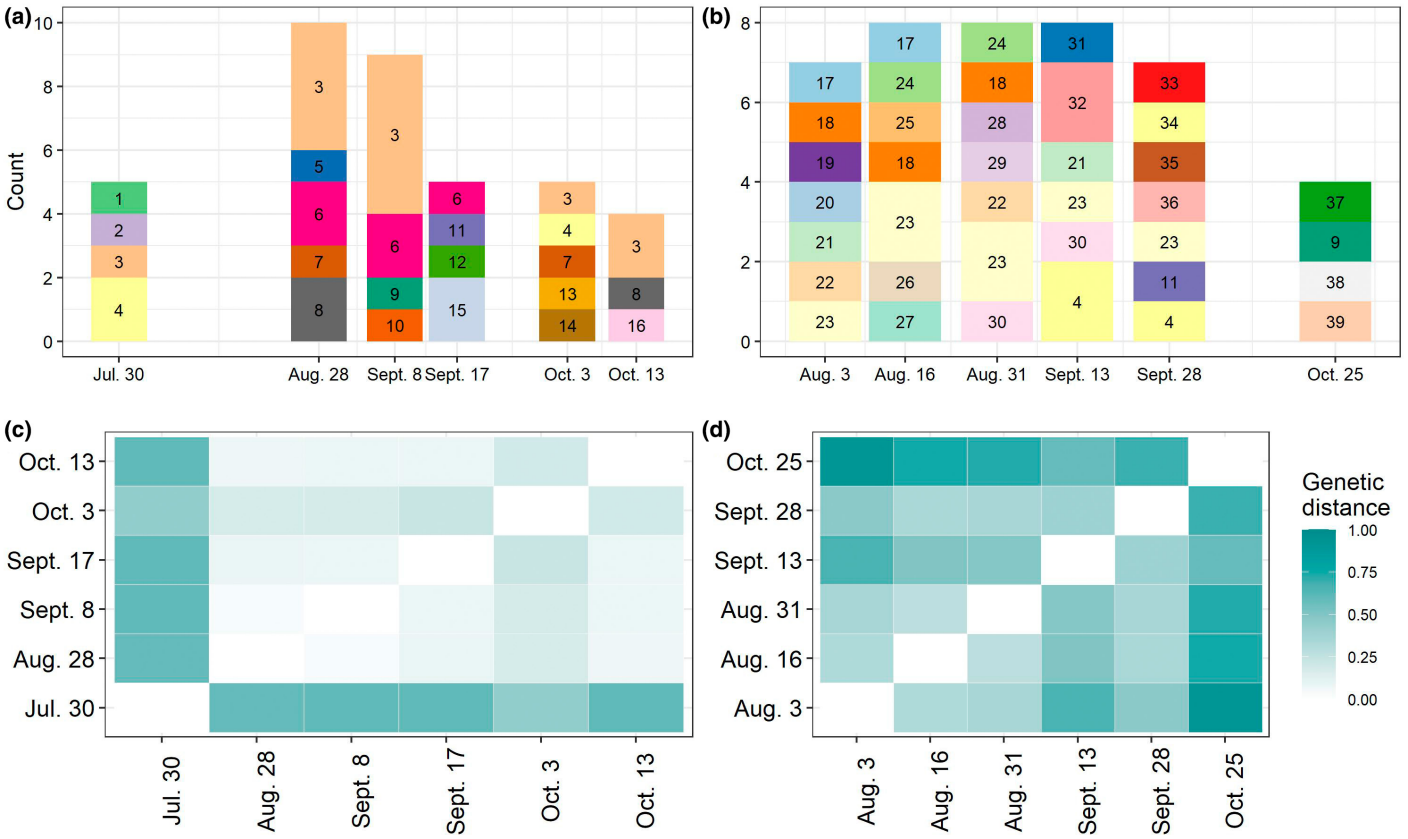
Many MLGs were identified at multiple time points throughout the epidemics in (a) Little Appleton and (b) Crooked, but, overall, the distance between *P. ramosa* populations increased with time. (c) For Little Appleton, this pattern is driven mostly by the difference between the genotypes at the beginning of the outbreak and those from later in the outbreak, whereas for Crooked (d), genetic distance between populations increased steadily with time between sampling dates. Genetic distance (c, d) is calculated between populations and is the average difference in allele frequencies between the populations summed over all loci (Prevosti et al., 1975).

For both outbreaks, there were multiple cases where a particular parasite genotype was identified on multiple sampling dates (Figure 2a,b), indicating persistence of some genotypes through time. Indeed, some genotypes were present throughout the entire sampling period (e.g., the genotype labeled “3” in panel C was found on all but one of the sampling dates, and genotype “4” was found in late July and early October). However, despite this persistence of some genotypes throughout the study, genetic distance between *P. ramosa* populations at different sampling dates increased with the time between sampling (Little Appleton: *F*_1,13_ = 8.41, *p* = .012; Crooked: *F*_1,13_ = 20.99, *p* < .001; Figure 2c,d). For parasites in Little Appleton, this change was driven mostly by the large genetic distance between genotypes at the beginning of the outbreak and genotype composition on the rest of the sampling dates (Figure 2c), whereas in Crooked, genetic distance between populations increased more steadily as time between sampling dates increased (Figure 2d). Interestingly, three genotypes (“4,” “9,” and “11” in Figure 2a,b) were found in both Little Appleton and Crooked.

## 4 DISCUSSION

We tracked *P. ramosa* genotypes through epidemics in two lakes, one with a large outbreak and one with a small outbreak. Parasite genetic diversity remained relatively constant over time, but, surprisingly, genetic diversity was much higher in the lake with the smaller outbreak (Crooked). We found signatures of evolution in both lakes: parasite genotypes structured by sample date in Little Appleton and the genetic distance between parasite populations increased with time between sampling dates in both outbreaks. In Little Appleton, there was a large change in genetic distance between the first sampling date and the second and relatively little change thereafter, whereas, in Crooked, the increase in genetic distance occurred more gradually over time. Overall, our data show that parasite evolutionary trajectories may differ across outbreaks and that ecological drivers and feedbacks associated with epidemic size should be more extensively explored. However, since only two outbreaks were explored over a single season, limited conclusions can be drawn about general patterns of parasite evolution from this study.

A priori, we would have expected greater diversity of *P. ramosa* in the larger outbreak, since there were presumably more opportunities for different *P. ramosa* genotypes to infect a large number of susceptible hosts. Why, then, did we see lower diversity in the much larger Little Appleton outbreak? One possibility is that selection might have been more efficient during this larger outbreak; in general, selection is more efficient in larger populations (Weber, 1990). The observed structuring of parasite genotypes by sampling date in Little Appleton might indicate that different parasite genotypes were selected over time perhaps due to changes in the host genotypes present or other ecological factors impacting parasite fitness. Indeed, host population structure has been documented to shift during other *P. ramosa* epidemics (Duncan & Little, 2007; Mitchell et al., 2004). Selection on parasites by abiotic conditions or by hosts can lead to local adaptation (Koskella, 2014; Lively et al., 2004) and negative frequency‐dependent selection (Ebert, 2008). Importantly, both phenomena have been observed in the *Daphnia*–*P. ramosa* system. *P. ramosa* assemblages are locally adapted to abiotic conditions (namely: light penetration into lakes; (Rogalski & Duffy, 2020)), and negative frequency‐dependent selection has been observed in the *Daphnia–P. ramosa* system over decadal time scales (Decaestecker et al., 2007) and has been implicated in other *Daphnia*–parasite systems from observations of *Daphnia* genotype turnover within epidemics (Turko et al., 2018; Wolinska & Spaak, 2009). Additional ecological factors such as predation could also influence parasite evolution in this system. It is noteworthy that Gowler et al. (2022) documented that *P. ramosa* evolved reduced spore production during this same epidemic in Little Appleton, potentially due to the selective pressure to shift from vegetative growth to the production of transmission spores earlier (thus generating fewer spores) in a likely high predation environment. Future studies that track whether epidemic size is generally associated with parasite genetic diversity, and whether those patterns are modulated by factors such as predation, would be very valuable, particularly since our study had only two populations.

Notably, the study lakes differed in host species (i.e., cladoceran) diversity with Crooked home to more *Daphnia* species than Little Appleton (M. A. Duffy, unpublished data). Species diversity is often correlated with genotypic diversity within species (Vellend & Geber, 2005). This could be important because diverse host populations often experience smaller epidemics (Ekroth et al., 2019; Gibson, 2021; King & Lively, 2012). Even if the diversity within our focal host did not differ between the two populations, the higher host species diversity in Crooked may have helped to minimize the parasite outbreak via a dilution effect (Hall et al., 2009; Keesing et al., 2006; Strauss et al., 2016). While different *Daphnia* species tend to become infected by distinct genotypes of *P. ramosa* (Duneau et al., 2011; Shaw, 2019), it is possible that these species can consume and kill parasite spores that infect *D. dentifera*. Future studies that track parasite evolution across several populations that vary in host diversity would help uncover the links between interspecific host diversity and genotypic diversity within parasite populations.

The parasite genotypes infecting at the beginning of outbreaks may influence parasite diversity and evolution for the remainder of epidemics (i.e., priority effects). In this system, epidemics are thought to begin when parasite spores from epidemics in previous years get resuspended from sediments (Decaestecker et al., 2004, 2007). The relative contribution to infections from spores from the spore bank vs. those produced in the ongoing epidemic is unknown, and this contribution likely changes through time and may depend on lake basin structure (Cáceres et al., 2006; Hall et al., 2010; Penczykowski et al., 2014). The pattern from Little Appleton could suggest that transmission from the sediments occurs at the beginning of an epidemic and after that, successful genotypes from the ongoing epidemic are amplified. The pattern from Crooked—where more different genotypes were found throughout the epidemic—may indicate instead that infection from the spore bank might continue throughout the season either due to feeding in the sediments or due to sediment resuspension into the water column. Future work that tracks the genotypes of free‐living spores in the water column, as well as the genotypes in infected hosts, would help determine the relative contributions of spores produced during an ongoing epidemic vs. those resuspended from sediment; ideally, this would be done in multiple populations that varied in the likelihood of spore resuspension (e.g., lakes that are weakly stratified vs. those with very strong stratification).

Our data also indicate that parasite genotypes might migrate between lakes as three genotypes were shared between Little Appleton and Crooked which are 9 miles (14.5 km) apart. This migration might be assisted by human recreational activities on lakes such as fishing, or by waterfowl, which can move parasite spores, infected hosts, and ephippia (Green & Figuerola, 2005); future studies tracking the genetic diversity of both hosts and parasites in multiple lakes would help uncover whether this is the case. However, it is also possible that, if we had more loci available, we would discover that these were, in fact, not the same genotype. While the number of loci that we used in this study was sufficient to detect substantial diversity within and between lakes, using newly discovered hypervariable regions of the *P. ramosa* genome (Andras et al., 2020) would likely uncover additional variation that was not captured by our VNTR analysis.

We quantified the genetic structure of populations of the parasite *P. ramosa* in infected *D. dentifera* hosts and found evidence of evolution within outbreaks. We hypothesize that intra‐ and interspecific host diversity, host population densities, epidemic size, and migration through time and space all influence the diversity and evolution of *P. ramosa* within epidemics. Future studies that include more epidemics and measure host genotypic diversity, as well as genotypes of spores in the water column, could help uncover the generality of these patterns and disentangle the mechanisms underlying this evolution.

## AUTHOR CONTRIBUTIONS

**Clara Shaw:** Conceptualization (lead); investigation (lead); data curation (lead); writing – original draft preparation (lead); writing – review and editing (equal). **Meghan Duffy:** Funding acquisition (lead); supervision (lead); writing – review and editing (equal).

## Supporting information


**Appendix S1.** Supporting Information

## Data Availability

The data that support the findings of this study are openly available at https://github.com/clarashaw/PasteuriaEvolution
