# Supplementary material for: Rapid evolution of a bacterial parasite during outbreaks in two Daphnia populations
Source: Ecol Evol. 2023 Jan 16;13(1):e9676. doi: 10.1002/ece3.9676 (PMC9843074; doi:10.1002/ece3.9676)
Supplement: Supplementary file 1 — Appendix S1. Supporting Information [file ECE3-13-e9676-s001.docx]

Appendix 1

**Table A1.** Forward and reverse primers used to genotype each VNTR locus. Forward primers all begin with the M13(-21) sequence (first 18 base pairs), allowing binding with a fluorescently labeled (6FAM or HEX) M13 primer (Schuelke 2000).

| Locus | Forward (5’-3’) | Reverse (5’-3’) |
| --- | --- | --- |
| Pr 1^1^ | TGTAAAACGACGGCCAGTACCTAAAGAACAGGAATATCTGGA | GCATGGAATGATTTTTGCTG |
| Pr 2^1^ | TGTAAAACGACGGCCAGTCTGCTGGATGGATGGACTACGTGA | ACCGGTCCCGTAGGTATAGG |
| Pr 3^1^ | TGTAAAACGACGGCCAGTGGACCAATCGAACCAGGTAT | AACGGTTTCTTCGCTTGTTG |
| Pr 4^1^ | TGTAAAACGACGGCCAGTGGTAACCCTGGATGTCCTGA | ATCCCGTTACAAATGGGACA |
| Pr 7^1^ | TGTAAAACGACGGCCAGTAACGTACTGACAAACCAAACCA | AATTTTTCTTAGATTGCTAGGTTG |
| Pr 11^1^ | TGTAAAACGACGGCCAGTCAAGCCAAATAAACGCATCC | TAGCGAAGAACACCAACGTG |
| Pr 12^1^ | TGTAAAACGACGGCCAGTTCTTTAGTAGTTGCTTTGCTTGAA | AACATCTTGGCACCCCTTTA |
| Pr 16^1^ | TGTAAAACGACGGCCAGTGGCAGGAACAAAAATTAAGCA | CGTTCCAAAGCGTTTTATGG |
| Pr 17^2^ | TGTAAAACGACGGCCAGTCACACACTTGCTCCATGGTC | AAACTAGATAGCGAAAAA |
| Pr 18^2^ | TGTAAAACGACGGCCAGTAAAGAAAGCTTCGTTTTAACGTG | CATTATCCACCCCCAAATCA |
| Pr 19^2^ | TGTAAAACGACGGCCAGTACGACCCAATCCGTTGATAG | CCAAGGCACGTTAGAAGAAA |

^1^Reported in (Mouton and Ebert 2008); ^2^Reported in (Andras and Ebert 2013).
